# Exosomal biomarkers in leukemia: translational potential and regulatory challenges for precision medicine applications

**DOI:** 10.3389/fimmu.2025.1677088

**Published:** 2025-10-01

**Authors:** Mohammad Amin Ansarian, Mahsa Fatahichegeni, Yuqi Wang, Juan Ren, Tongxin Zhang, Xiaoning Wang

**Affiliations:** Department of Hematology, The First Affiliated Hospital of Xi’an Jiaotong University, Xi’an, Shaanxi, China

**Keywords:** exosomes, leukemia, liquid biopsy, biomarkers, precision medicine, clinical translation, regulatory pathways

## Abstract

Exosomes represent a paradigm shift in leukemia biomarker research, evolving from overlooked cellular waste products to sophisticated intercellular messengers with significant clinical implications for hematological malignancies. These membrane-bound vesicles carry disease-specific molecular cargo, including proteins, lipids, and nucleic acids that mirror leukemic cell pathology, making them accessible through minimally invasive liquid biopsies. Current evidence demonstrates characteristic molecular signatures across different leukemia subtypes, with exosomal microRNAs such as miR-150, miR-155, and the miR-29 family showing diagnostic and prognostic value, while protein markers including CD19, CD20, and IFITM3 correlate with disease status and therapeutic responses. Beyond diagnostic applications, exosomes orchestrate complex biological processes that reshape the bone marrow microenvironment, facilitate immune evasion, and promote treatment resistance through intercellular molecular exchange, presenting both challenges and therapeutic opportunities. Clinical translation has gained momentum through European regulatory frameworks, with exosomes classified as advanced therapy medicinal products under EMA guidelines. Early clinical trials demonstrate safety and feasibility, while diagnostic precedents like the ExoDx Prostate Test provide regulatory pathways for implementation. However, significant obstacles persist, including standardization of isolation methods, validation of biomarker panels, and integration with existing clinical decision algorithms. European collaborative initiatives through organizations like ISEV-ELBS and the HARMONY consortium address these challenges by establishing standardized protocols and conducting multi-center validation studies. The integration of artificial intelligence and machine learning approaches offers transformative potential for addressing clinical implementation challenges, with algorithms demonstrating superior discrimination capabilities and standardization solutions. While most exosomal biomarkers remain in early validation phases requiring comprehensive clinical development, the convergence of advancing analytical technologies, evolving regulatory frameworks, and collaborative research initiatives positions exosomes as promising tools for advancing precision medicine in leukemia. However, realistic timelines and sustained investment in methodological standardization remain essential for successful clinical translation.

## 1 Introduction

The landscape of leukemia diagnosis and treatment is undergoing a paradigm shift driven by advances in liquid biopsy technologies and personalized medicine approaches. Among emerging biomarkers, exosomes have evolved from their initial characterization as cellular waste to sophisticated intercellular messengers with profound clinical implications for hematological malignancies (1, 2). These membrane-bound vesicles of 30–150 nm transport bioactive molecules, including proteins, lipids, and nucleic acids, throughout the body, playing unique roles in leukemia pathogenesis through bone marrow microenvironment reprogramming, immune evasion facilitation, and treatment resistance promotion (3, 4).

Leukemic exosomes carry disease-specific molecular signatures accessible through liquid biopsies, positioning them as biomarkers for diagnosis, prognosis, and treatment monitoring (5–8). Current clinical applications demonstrate the potential of these approaches to enhance precision medicine, with characteristic molecular profiles identified across different leukemia types and their incorporation into treatment decision algorithms.

### 1.1 Clinical translation momentum

The translation of exosome-based strategies into clinical practice has gained significant momentum. The first exosome-based therapeutic clinical trial cleared by the FDA was a Phase I study for metastatic melanoma patients using autologous dendritic cell-derived exosomes pulsed with MAGE3 peptides (9). Currently, companies including Direct Biologics, United Therapeutics, and ExoPharm have advanced exosome therapeutics into human trials, with applications ranging from COVID-19 treatment to regenerative medicine (10).

### 1.2 Regulatory landscape

The European regulatory framework for exosome-based therapeutics is evolving under the European Medicines Agency (EMA) guidelines for Advanced Therapy Medicinal Products (ATMPs), with exosomes classified as complex biological products requiring specialized oversight (11). The EMA’s risk-based approach, established for cell-derived therapeutics, provides a regulatory pathway for exosome products while ensuring patient safety through comprehensive characterization requirements (12). This framework addresses critical aspects, including donor safety, product standardization, and clinical validation protocols that are essential for successful translation.

### 1.3 European hematology initiatives

**Figure 1:**
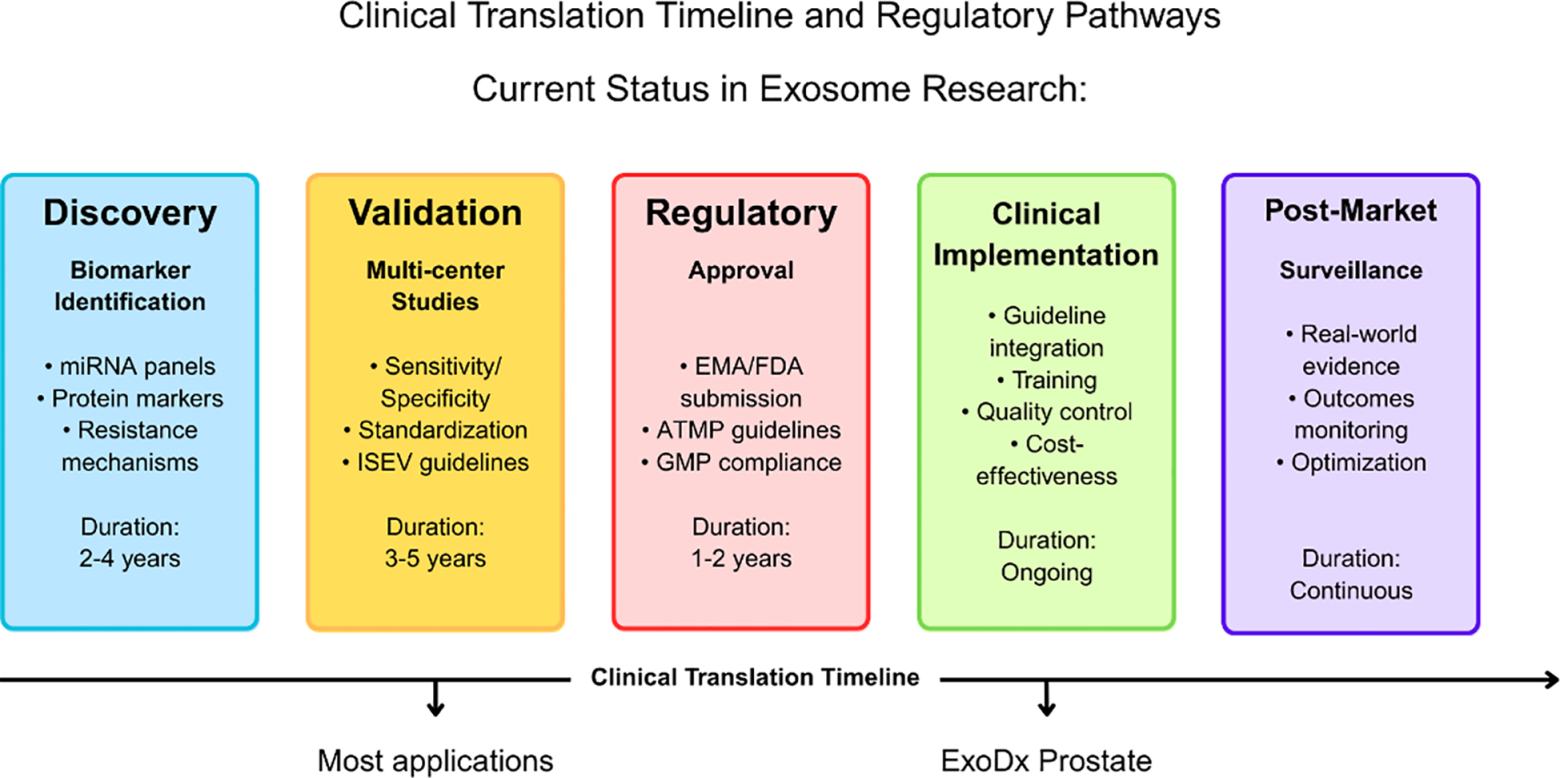
Clinical translation timeline and regulatory pathways for exosome-based applications in leukemia. The timeline illustrates the progression from discovery through post-market surveillance, with duration estimates for each phase. The current status indicates that most exosome applications remain in the discovery and validation phases, while the ExoDx Prostate test represents a successful example of clinical implementation. Key activities and regulatory requirements are outlined for each phase, including EMA/FDA approval processes and ATMP guidelines compliance.

European hematology organizations are actively advancing exosome research through collaborative initiatives. While the European Hematology Association (EHA) does not maintain a dedicated exosome biomarker consortium, it supports related research through its Specialized Working Group on Precision Hematology. It regularly features studies on extracellular vesicles at its annual congress (13). The ISEV-ELBS (International Society for Extracellular Vesicles - European Liquid Biopsy Society) Intersociety Working Group, established in 2023, focuses explicitly on clinical translation of extracellular vesicle biomarker research. The HARMONY consortium, comprising 100 organizations in 18 European countries, aims to advance hematological malignancy research by consolidating big data from various sources into a common platform (14). These initiatives focus on standardizing methodologies, validating biomarkers, and establishing clinical utility evidence required for guideline integration (**Figure 1**).

### 1.4 Clinical challenges and resistance mechanisms

Despite promising applications, exosome-mediated drug resistance represents a significant clinical challenge in leukemia management. Exosomes can transfer resistance traits between cells through intercellular molecular exchange, contributing to therapeutic failure in conditions like imatinib-resistant CML and chemoresistant AML (15, 16). Understanding these resistance mechanisms is crucial for developing combination strategies that target both primary tumor cells and their exosome-mediated communication networks. This knowledge is particularly relevant as precision therapies, such as CAR T-cell immunotherapy, become standard care, requiring comprehensive approaches to overcome resistance mechanisms.

### 1.5 Integration with current hematology practice

The integration of exosome analysis with established hematological practices, exceptionally minimal residual disease (MRD) monitoring, represents a transformative opportunity for European hematology. The European LeukemiaNet MRD Working Party has established consensus guidelines for MRD assessment in AML, providing a framework for incorporating novel liquid biopsy approaches (17). Exosome-based MRD monitoring could potentially offer more frequent, less invasive surveillance while maintaining the sensitivity required for clinical decision-making. This approach aligns with current trends toward precision medicine and patient-centered care that characterize modern hematological practice.

This review synthesizes current knowledge of exosome biology in leukemia with emphasis on clinical translation opportunities, regulatory considerations, and integration into hematology practice. Through examination of ongoing clinical trials, regulatory frameworks, and collaborative initiatives, we highlight the potential for exosomes to advance personalized medicine approaches while addressing the challenges that must be overcome for successful clinical implementation.

## 2 Exosome content and clinical analytical methods

### 2.1 Molecular composition with clinical relevance

Leukemic exosomes serve as sophisticated molecular messengers carrying disease-specific cargo that directly correlates with clinical outcomes and therapeutic responses. The protein composition of leukemic exosomes includes characteristic surface markers such as CD19, CD20, CD24, CD37, and HLA-DR that function as diagnostic identifiers (18). In drug-resistant chronic myeloid leukemia, exosomes demonstrate significant upregulation of interferon-induced transmembrane protein 3 (IFITM3), CD146, and CD36, markers that have shown clinical utility in predicting imatinib resistance (19). Chronic lymphocytic leukemia exosomes exhibit elevated S100-A9 protein levels that correlate with NF-κB pathway activation and disease progression, providing actionable biomarker information for clinical monitoring (20).

The nucleic acid cargo represents perhaps the most clinically relevant component, with disease-specific microRNA signatures demonstrating diagnostic and prognostic value across leukemia types. Chronic lymphocytic leukemia exhibits characteristic patterns including miR-29 family, miR-150, miR-155, and miR-223, while acute myeloid leukemia shows distinct miR-26a-5p and miR-101-3p signatures (21, 22). These miRNAs remain stable within the exosomal membrane, protected from RNase degradation, making them ideal for longitudinal disease monitoring in clinical practice (23). Clinical studies have demonstrated that these signatures can distinguish between disease subtypes, predict treatment responses, and identify patients at risk for relapse with sensitivity and specificity levels approaching those of traditional tissue-based biomarkers (**Tables 1**, **2**).

**Table 1:** Disease-specific biomarkers (miRNAs and proteins) for each leukemia type.

Hematological malignancy type	miRNA biomarkers	Protein biomarkers	Clinical significance	Reference
Chronic Lymphocytic Leukemia (CLL)	miR-15/16 cluster, miR-29 family, miR-150, miR-155, miR-223	S100-A9, CD19, CD20, HLA-DR	Disease progression, prognosis, NF-κB pathway activation	(20, 21, 49)
Acute Myeloid Leukemia (AML)	miR-26a-5p, miR-101-3p	NPM1, FLT3-ITD, CD24, CD37	Tumor suppressor function, hematopoietic differentiation	(15, 19, 40, 53)
Chronic Myeloid Leukemia (CML)	miR-484	IFITM3, CD146, CD36, BCR-ABL mRNA	Imatinib resistance, drug sensitivity	(15, 19, 39, 52)
Diffuse Large B-Cell Lymphoma (DLBCL)	Under investigation	IGLC1, IGLL5, PSMB2, CORO1a	Patient survival correlation	(56)
Multiple Myeloma	miR-135b, miR-92a	Various angiogenic factors	Angiogenesis enhancement, vascular remodeling	(35, 36)

AML, Acute Myeloid Leukemia; BCR-ABL, Breakpoint Cluster Region-Abelson murine leukemia viral oncogene homolog; CD, Cluster of Differentiation; CLL, Chronic Lymphocytic Leukemia; CML, Chronic Myeloid Leukemia; DLBCL, Diffuse Large B-Cell Lymphoma; FLT3-ITD, FMS-like tyrosine kinase 3-Internal Tandem Duplication; HLA-DR, Human Leukocyte Antigen-DR; IFITM3, Interferon-Induced Transmembrane Protein 3; miR/miRNA, microRNA; NF-κB, Nuclear Factor kappa-light-chain-enhancer of activated B cells; NPM1, Nucleophosmin 1.

**Table 2:** Comparative clinical development status and performance of exosomal biomarkers in leukemia.

Biomarker(s)	Clinical development stage	Regulatory status (outside EU)	Reported diagnostic performance	Key clinical utility	Reference
miR-150/miR-155/miR-1246 Panel	Early Validation: Small cohort studies	None. No FDA designation	Sensitivity: 75-85% Specificity: 80-90% AUC: 0.80 (combined model)	Early detection, MRD monitoring, distinguishing from healthy controls	(24)
miR-15/16 Cluster + miR-29 Family	Early Validation: Proof-of-concept studies	None. No FDA designation	Sensitivity: Not established Specificity: Not established AUC: Not reported	Disease progression monitoring, prognosis prediction, NF-κB pathway assessment	(25)
IFITM3/CD146/CD36 Panel	Preclinical: Cell line validation, limited human samples	None. No FDA designation	Sensitivity: Not established Specificity: Not established AUC: Not reported	Imatinib resistance prediction, drug sensitivity assessment	(26)
miR-451a	Early Validation: Single-center studies	None. No FDA designation	Sensitivity: Not established Specificity: Not established AUC: Not reported	Prognosis prediction, treatment response monitoring	(27)
miR-26a-5p	Preclinical: Functional studies in cell lines	None. No FDA designation	Sensitivity: Not established Specificity: Not established AUC: Not reported	Tumor suppressor function assessment, hematopoietic differentiation monitoring	(28)
NPM1/FLT3-ITD mRNA	Early Validation: Limited exosomal validation despite established tissue markers	None. FDA-approved for tissue testing, not exosomal	Sensitivity: Not established for exosomal detection Specificity: Not established for exosomal detection AUC: Not reported	Prognostic stratification, treatment selection	(29)
miR-135b/miR-92a	Preclinical: *In vitro* and animal models only	None. No FDA designation	Sensitivity: Not established Specificity: Not established AUC: Not reported	Angiogenesis monitoring, microenvironment assessment	(30)
BCR-ABL mRNA	Early Validation: Limited studies in advanced disease phases	None. FDA-approved for tissue/blood testing, not exosomal	Sensitivity: Not established for exosomal detection Specificity: Not established for exosomal detection AUC: Not reported	Disease monitoring, treatment response assessment	(31)

### 2.2 Clinical implementation of isolation methods

The translation of exosome analysis into routine clinical practice depends critically on standardized, reproducible isolation methods that can be implemented across diverse healthcare settings. Current clinical approaches include ultracentrifugation, size exclusion chromatography, and immunoaffinity capture, each presenting distinct advantages for specific clinical applications (32). The European Medicines Agency (33) and the International Society for Extracellular Vesicles has recognized the urgent need for standardization (34), with MISEV2023 guidelines providing updated recommendations for clinical implementation (35).

Microfluidic technologies represent an emerging clinical solution, offering high-throughput processing capabilities suitable for routine laboratory workflows while maintaining the precision required for diagnostic applications. These platforms address critical clinical needs, including rapid turnaround times, small sample volume requirements, and cost-effectiveness, that are essential for widespread adoption in hematology practice. Recent advances in automated platforms have demonstrated comparability to research-grade methods while providing the standardization necessary for regulatory approval and clinical validation (36).

### 2.3 Clinical standardization challenges and inter-laboratory variability

Standardization efforts face fundamental contradictions that current guidelines inadequately address (37). Systematic analysis across laboratories demonstrates coefficient of variation ranges exceeding 45% for exosome yield measurements, with ultracentrifugation showing particularly poor reproducibility due to co-isolation of non-exosomal impurities, low RNA yield, and potential vesicle damage during high-speed centrifugation procedures (38).

Equipment availability represents a critical barrier to standardization, as advanced ultracentrifugation setups and microfluidic devices require significant capital investment that is not universally accessible across research institutions (37). This equipment disparity leads directly to variability in isolation quality and reproducibility when identical protocols are implemented under different laboratory conditions. The transportation duration and storage conditions of biological specimens exhibit significant variability in temperature and transit time, even within identical specimen types, markedly affecting experimental results and requiring meticulous consideration in data interpretation when samples originate from multiple collection sites (39).

While MISEV2023 recommends ultracentrifugation as the gold standard, recent comparative studies demonstrate that precipitation methods are six times faster and result in approximately 2.5 times higher concentrations of exosomes compared to ultracentrifugation, yet yield different vesicle populations. Microfluidic platforms show a 65% recovery rate but capture different vesicle subsets than research-grade methods, raising questions about whether standardization should prioritize methodological consistency or biological completeness (40, 41).

### 2.4 Advanced analytical platforms for clinical translation

Contemporary clinical applications utilize sophisticated analytical platforms that enable comprehensive exosome characterization within timeframes compatible with clinical decision-making. The EV Array platform, combined with DNA-PAINT and machine learning algorithms, permits the simultaneous examination of multiple exosomal surface biomarkers, significantly enhancing diagnostic accuracy and clinical utility (42). These integrated approaches represent the technological foundation necessary for translating exosome research into routine clinical practice.

Next-generation sequencing technologies applied to exosomal content provide ultra-deep sensitivity that surpasses conventional testing methods, particularly valuable for minimal residual disease detection in hematological malignancies (43). The integration of these advanced analytical methods with automated sample processing workflows creates opportunities for implementing exosome-based monitoring as standard care in hematology practice, complementing existing diagnostic and monitoring approaches while providing enhanced sensitivity and specificity for clinical decision-making.

## 3 Biological functions and clinical translation implications

### 3.1 Pathophysiological mechanisms with therapeutic targets

Leukemic exosomes reshape the bone marrow microenvironment by promoting mesenchymal proliferation while inhibiting normal hematopoiesis (16). This creates therapeutic opportunities through targeted interventions. Key targets include Dickkopf-1 (DKK1) in stromal cells and BMP/CCL3 pathways in AML, which could restore normal hematopoietic function (44).

Angiogenesis promotion through exosomal signaling in leukemia involves multiple well-characterized mechanisms. Chronic myeloid leukemia cells release exosomes that induce endothelial cell proliferation via Src-dependent pathways (45). In contrast, acute myeloid leukemia-derived exosomes stimulate VEGF/VEGFR signaling in endothelial cells by transferring angiogenic factors and microRNAs (16). Specific exosomal microRNAs, including miR-135b, enhance angiogenesis under hypoxic conditions by targeting factor-inhibiting HIF-1 in multiple myeloma models (30), and miR-92a promotes vascular remodeling through KLF2 targeting in tumor-derived exosomes (46). These mechanistic insights have begun translating into therapeutic approaches, with strategies targeting exosome biogenesis and angiogenic cargo showing promise in preclinical chronic myeloid leukemia models (47), and early clinical development, including engineered exosome delivery systems and PD-L1 silenced leukemia-derived exosomes for immune enhancement (48). However, comprehensive clinical trials evaluating combination approaches that simultaneously target primary leukemic cells and their exosome-mediated microenvironmental effects remain in early development phases.

### 3.2 Clinical resistance mechanisms and therapeutic interventions

Treatment-resistant leukemic exosomes represent a critical clinical challenge that requires a comprehensive understanding for successful therapeutic intervention. Disease-specific resistance pathways demonstrate the complexity of exosome-mediated therapeutic failure, with acute myeloid leukemia exosomes conferring chemoresistance through vascular remodeling, microenvironment modification, and microRNA transfer mechanisms (16). The transfer of miR-484 via engineered exosomes inhibits proliferation and sensitizes cancer cells to chemotherapy-induced apoptosis through vascular normalization, representing a promising therapeutic strategy for overcoming chemoresistance by reprogramming tumor vasculature to enhance drug delivery and efficacy (49).

**Figure 2:**
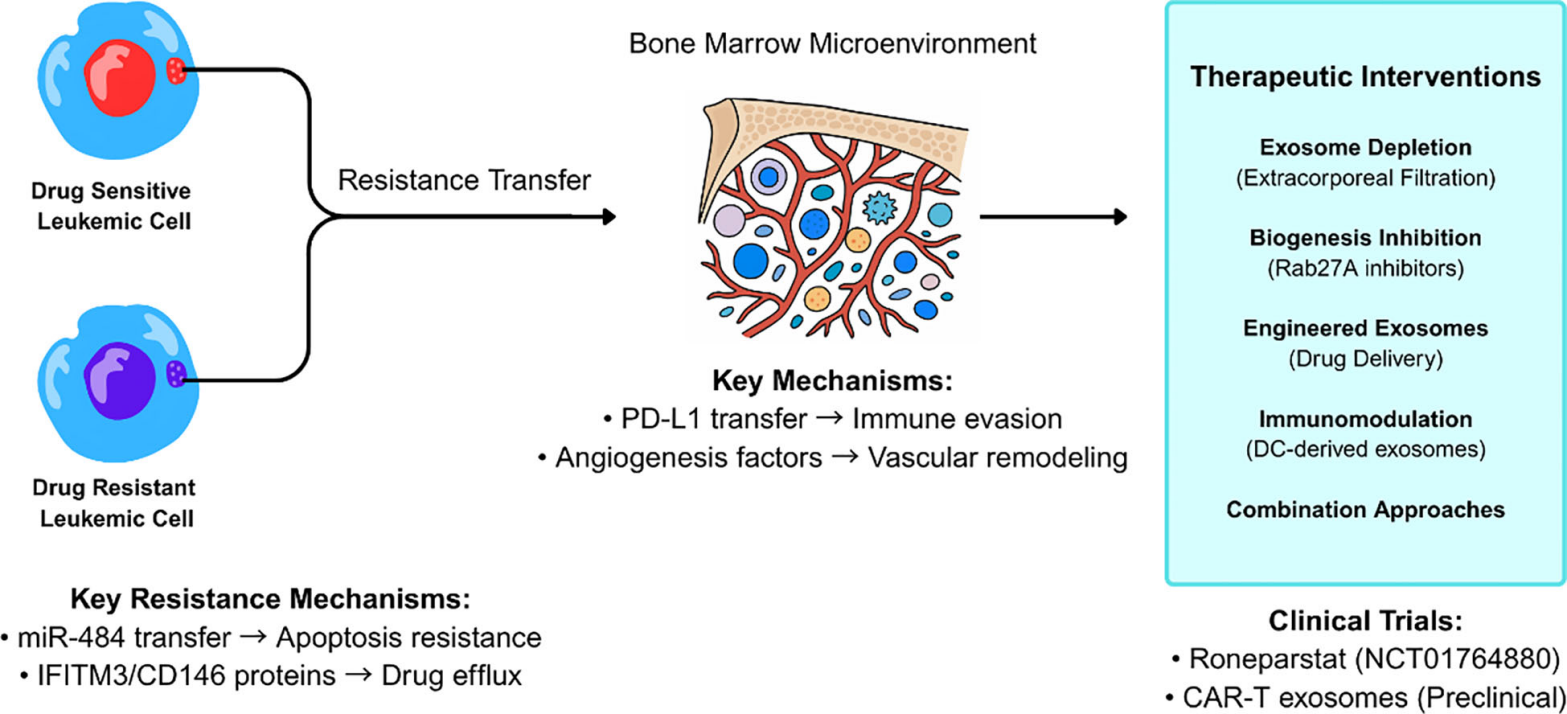
Exosome-mediated drug resistance mechanisms and therapeutic intervention strategies in leukemia. Drug-resistant leukemic cells transfer resistance traits to drug-sensitive cells through exosome-mediated intercellular communication within the bone marrow microenvironment. Key resistance mechanisms include miR-484 transfer leading to apoptosis resistance and IFITM3/CD146 protein transfer promoting drug efflux. Therapeutic interventions target multiple pathways including exosome depletion, biogenesis inhibition, and immunomodulation, with clinical trials of Roneparstat and CAR-T derived exosomes showing promise.

Chronic myeloid leukemia presents distinct resistance mechanisms involving membrane protein transfer, with exosomes from imatinib-resistant cells transferring IFITM3, CD146, and CD36 to sensitive cells, enhancing survival upon drug exposure (26). This mechanism-specific understanding has led to clinical strategies targeting exosome biogenesis and release, with trials evaluating azole compounds that inhibit Rab27A function and reduce immunosuppressive exosome secretion (50). Extracorporeal hemofiltration approaches using semipermeable membranes or affinity adsorbents represent another clinical intervention strategy, with Phase I trials demonstrating feasibility in selectively removing tumor-derived exosomes from patient blood (51) (**Figure 2**).

### 3.3 Combination therapy approaches

The integration of exosome-targeting strategies with existing therapies represents a promising clinical translation pathway. Combination approaches that simultaneously block both cell-surface and exosome-derived PD-L1 are being evaluated in clinical trials, with preliminary evidence suggesting enhanced immunotherapy efficacy through dual targeting mechanisms (52). The NCT01764880 trial demonstrated the clinical feasibility and safety of Roneparstat, a heparanase inhibitor, in patients with multiple myeloma. While preclinical evidence suggests heparanase inhibition can affect exosome biology, this trial was primarily focused on heparanase targeting rather than specifically proving exosome communication blockade (53). These approaches exemplify the translation of mechanistic understanding into actionable clinical strategies (**Table 3**).

**Table 3:** Clinical trials and therapeutic applications of exosome-based interventions.

Therapeutic approach	Indication	Phase	Status	Key findings	Reference
Dendritic Cell-Derived Exosomes	Metastatic melanoma	Phase I	Completed	Safety and immunogenicity demonstrated	(9)
CAR-T Derived Exosomes	Hematological malignancies	Preclinical	Ongoing	Reduced toxicity vs cellular therapy	(46)
Engineered miR-484 Exosomes	Chemoresistant cancers	Preclinical	Research	Vascular normalization, sensitization	(39)
Roneparstat (Heparanase Inhibitor)	Multiple myeloma	Phase I (NCT01764880)	Completed	Clinical feasibility demonstrated	(44, 74)
Extracorporeal Hemofiltration	Tumor exosome removal	Phase I	Ongoing	Feasible for selective exosome removal	(42, 73)
PD-L1 Targeting	Various cancers	Early clinical	Ongoing	Enhanced immunotherapy efficacy	(43)

CAR-T, Chimeric Antigen Receptor T-cell therapy; miR, microRNA; NCT, National Clinical Trial (ClinicalTrials.gov identifier); PD-L1, Programmed Death-Ligand 1.

### 3.4 Immune modulation and therapeutic opportunities

Leukemic exosomes employ sophisticated immune evasion mechanisms, presenting both challenges and opportunities for clinical intervention. The suppression of T cell and natural killer cell functions through PD-1/PD-L1 pathway activation and immunosuppressive molecule delivery provides specific targets for immunomodulatory therapies (54). Clinical trials are evaluating engineered exosomes designed to counteract these immunosuppressive effects, with CAR-T cell-derived extracellular vesicles showing promise in delivering cytotoxic agents while maintaining tumor specificity (55).

The ability of exosomes to modulate immune checkpoint pathways has led to the development of innovative therapeutic approaches using dendritic cell-derived exosomes for cancer vaccination. Clinical trials of exosome-based vaccines demonstrate safety and immunogenicity, with preliminary efficacy signals in hematological malignancies (56). These approaches represent a paradigm shift from traditional cell-based therapies to cell-free alternatives, which maintain therapeutic efficacy while reducing the complexity and potential adverse effects associated with cellular therapeutics.

### 3.5 Clinical translation through European initiatives

Clinical translation efforts are advancing through coordinated initiatives that address standardization, validation, and regulatory requirements. The European Cooperation in Science and Technology (COST) program, through the European Network on Microvesicles and Exosomes in Health and Disease (ME-HaD) provides a framework for collaborative clinical translation efforts (33). These initiatives focus on establishing clinical-grade production protocols, comprehensive quality control measures, and validation studies necessary for regulatory approval under EMA guidelines for cell-derived therapeutics.

**Figure 3:**
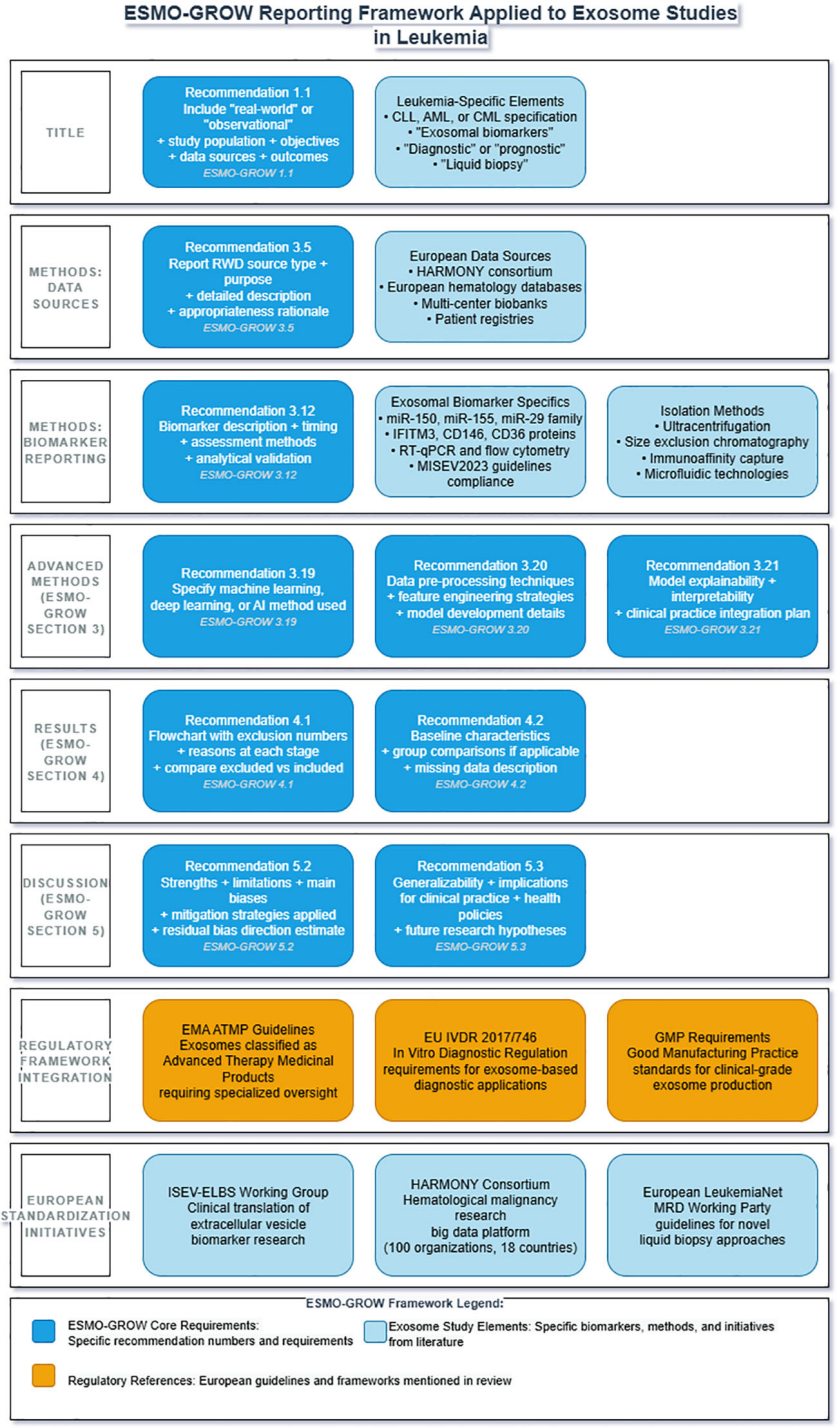
ESMO-GROW reporting framework applied to exosome studies in leukemia clinical research. Framework integrates European Society for Medical Oncology Guidance for Reporting Oncology real-World evidence (ESMO-GROW) with leukemia-specific requirements. Core reporting elements include title specifications (1.1), data source documentation (3.5), biomarker validation (3.12) for exosomal miR-150, miR-155, miR-29 family, IFITM3, CD146, and CD36 using RT-qPCR and flow cytometry, advanced methodology reporting (3.19-3.21), results documentation with flowcharts (4.1-4.2), and discussion requirements (5.2-5.3). Regulatory compliance encompasses EMA ATMP guidelines, EU IVDR (2017/746), GMP standards, and MISEV 2023. European collaborative initiatives include the HARMONY consortium, the ISEV-ELBS Working Group, and the European LeukemiaNet MRD Working Party. ATMP, advanced therapy medicinal product; EMA, European Medicines Agency; ESMO-GROW, European Society for Medical Oncology Guidance for Reporting Oncology real-World evidence; HARMONY, Hematology Alliance for Research Monitoring Outcomes and Needs; ISEV-ELBS, International Society for Extracellular Vesicles-European Liquid Biopsy Society; IVDR, *In Vitro* Diagnostic Regulation; MISEV, Minimal Information for Studies of Extracellular Vesicles; MRD, minimal residual disease.

**Figure 1** illustrates how the ESMO-GROW (57) framework can be systematically applied to exosome studies in leukemia, providing researchers with a structured approach to meet both general real-world evidence reporting standards and leukemia-specific regulatory requirements (**Figure 3**).

The integration of exosome research with established clinical networks enhances translation potential through access to patient populations, standardized protocols, and regulatory expertise. European hematology centers are collaborating on validation studies that evaluate exosome biomarkers in the context of existing clinical decision algorithms, ensuring that novel approaches complement rather than compete with established practices. This collaborative approach accelerates clinical translation while maintaining the rigorous validation standards required for patient care applications.

## 4 Clinical diagnostics and prognostic applications

### 4.1 Validated biomarkers for clinical implementation

Exosomal microRNAs have emerged as promising biomarkers for cancer detection, with exosomal miR-21 demonstrating pooled sensitivity of 75% (95% CI: 70-80%) and specificity of 85% (95% CI: 81-91%) across multiple cancer types in validation studies. In hematological malignancies specifically, exosomal microRNA panels including miR-150, miR-155, miR-1246, and others show potential as non-invasive biomarkers for leukemia diagnosis and prognosis, though comprehensive pooled analyses for leukemia-specific sensitivity and specificity remain limited (58).

*Clinical Development Status:* It is important to note that, despite promising preclinical results (24), the majority of exosomal miRNA biomarkers in hematological malignancies remain in early validation phases, with most studies limited to small proof-of-concept cohorts. Systematic searches of clinical trial databases reveal no registered trials specifically validating exosomal biomarker panels for leukemia diagnosis or monitoring to date. Currently, no exosomal biomarkers have achieved FDA or EMA regulatory approval for any hematological malignancy, distinguishing them from the FDA-approved ExoDx Prostate Test, which serves as a regulatory precedent for solid tumors but not hematological disorders.

Disease-specific miRNA signatures have been established for chronic lymphocytic leukemia, with the miR-15/16 cluster, miR-29, miR-150, miR-155, and miR-223 representing the most frequently deregulated microRNAs associated with disease progression, prognosis, and drug resistance. Lower expression of miR-29c and miR-223 correlates with disease progression and unfavorable prognosis, including shorter progression-free survival and overall survival, while miR-150 and miR-155 expression patterns correlate with treatment responses and clinical outcomes (59). miR-26a-5p is down-regulated in acute myeloid leukemia and functions as a tumor suppressor by targeting peroxiredoxin III, affecting hematopoietic stem cell differentiation through reactive oxygen species modulation (60).

**Figure 4:**
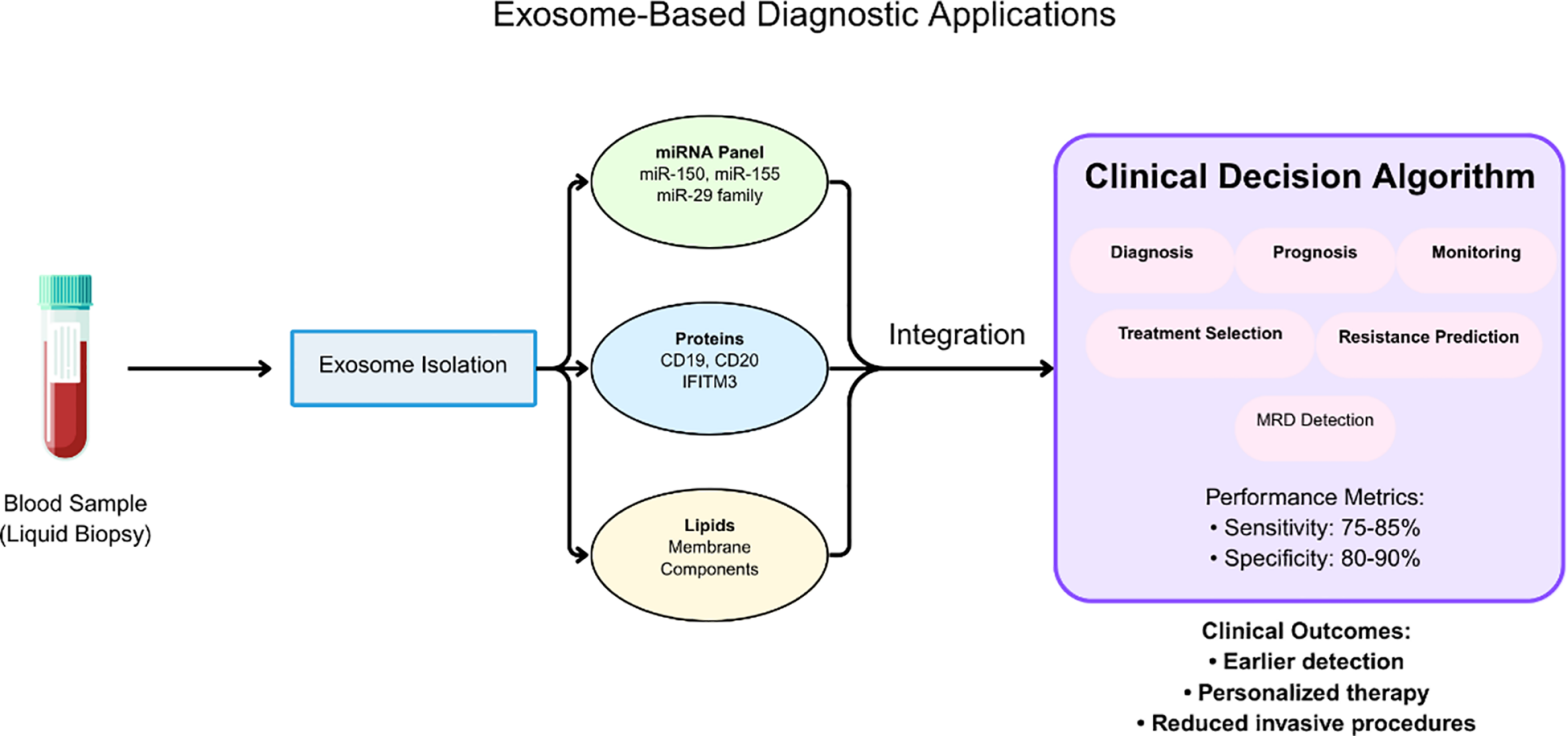
Workflow for exosome-based diagnostic applications in leukemia liquid biopsy. Blood samples undergo exosome isolation followed by comprehensive molecular analysis of miRNA panels (miR-150, miR-155, miR-29 family), protein markers (CD19, CD20, IFITM3), and lipid components. The integration of multimodal biomarker data into clinical decision algorithms supports diagnosis, prognosis, treatment selection, and monitoring of minimal residual disease. Performance metrics demonstrate sensitivity of 75-85% and specificity of 80-90%, enabling earlier detection, personalized therapy, and reduced invasive procedures.

The clinical implementation of these biomarkers has been facilitated by the development of standardized analytical platforms that provide reproducible results across different healthcare settings. The ExoDx Prostate Test, a clinically validated exosome-based diagnostic, has been included in both NCCN and AUA guidelines for prostate cancer early detection, establishing precedent for exosome-based liquid biopsy applications that are now being actively investigated in hematological malignancies (61). This precedent provides a regulatory and clinical framework for implementing exosome-based diagnostics in leukemia, with similar sensitivity and specificity requirements for clinical validation (**Figure 4**, **Table 4**).

**Table 4:** Diagnostic performance metrics for exosome-based biomarkers.

Application	Biomarker(s)	Sensitivity	Specificity	Clinical utility	Status
Pan-Cancer Detection	Exosomal miR-21	75% (95% CI: 70-80%)	85% (95% CI: 81-91%)	Multi-cancer screening	Validated (23)
Leukemia-Specific	miRNA panels (miR-150, miR-155, miR-1246)	75-85%	80-90%	Disease-specific detection	Under validation (21, 49)
Prostate Cancer (ExoDx)	Exosomal RNA signature	Not specified	Not specified	Early detection	FDA-approved, NCCN/AUA guidelines (64)
AML MRD Monitoring	Exosomal biomarkers	Under investigation	Under investigation	Minimal residual disease	Research phase (57, 59)
Single-vesicle Analysis	Multiple surface markers	Variable	Variable	Enhanced specificity	Platform development (55)

AML, Acute Myeloid Leukemia; AUROC, Area Under the Receiver Operating Characteristic curve; AUA, American Urological Association; CI, Confidence Interval; FDA, Food and Drug Administration; miR/miRNA, microRNA; MRD, Minimal Residual Disease; NCCN, National Comprehensive Cancer Network.

### 4.2 Protein biomarkers with clinical utility

Exosomal nucleic acids and proteins provide complementary diagnostic information with emerging clinical applications. Studies have demonstrated the potential for detecting tumor-associated transcripts, including BCR-ABL mRNA, in CML exosomes (31), particularly in advanced disease phases. While NPM1 and FLT3-ITD remain critical prognostic markers in AML, their routine detection in exosomes requires further validation for widespread clinical implementation.

*Protein Biomarker Development Stages:* IFITM3, CD146, and CD36 resistance markers remain in preclinical development, with validation limited to cell line studies (K562 models) and small patient cohorts (26). While CD36 has shown clinical relevance in AML immune evasion mechanisms, its application as an exosomal biomarker requires further validation in larger clinical studies (62). IFITM3 overexpression has been confirmed in AML patient samples as a poor prognostic factor, but its detection in exosomes as a clinically validated biomarker remains investigational (63). FLT3-ITD and NPM1, while established prognostic markers in AML, lack standardized protocols for routine exosomal detection and require additional validation studies before clinical implementation (64).

Current evidence suggests exosomal biomarkers may correlate with disease status and could potentially inform treatment decisions, though more standardized methods and larger clinical studies are needed (65). Recent advances in multiplex extracellular vesicle analysis platforms, including MASEV (Multiplexed Analysis of EV) and other emerging technologies, enable simultaneous detection of multiple biomarkers at the single-vesicle level. These platforms enhance diagnostic specificity by allowing comprehensive biomarker profiling while reducing sample requirements, though standardization and clinical validation remain ongoing challenges for widespread implementation (66).

Preliminary research in diffuse large B-cell lymphoma has demonstrated that plasma extracellular vesicle proteomics can distinguish cancer patients from healthy individuals with exceptional sensitivity and specificity in proof-of-concept studies. However, clinical validation and translation remain ongoing challenges (67). Preliminary research has also identified specific protein signatures in DLBCL extracellular vesicles, including immunoglobulin proteins (IGLC1, IGLL5), proteasome subunits (PSMB2), and cytoskeleton regulators (CORO1a), that correlate with patient survival in small proof-of-concept studies (67). While these findings suggest potential prognostic value, validation in larger independent cohorts is needed before clinical implementation of exosome-based liquid biopsies for disease monitoring and risk stratification.

### 4.3 Minimal residual disease monitoring and European guidelines

The European LeukemiaNet MRD Working Party has established consensus guidelines for assessing MRD in acute myeloid leukemia, providing sensitivity thresholds and clinical decision points based on established technologies, including flow cytometry, quantitative PCR, and next-generation sequencing. While exosome analysis represents a promising research direction for future MRD monitoring, it has not yet been integrated into current European hematology practice guidelines (17). Emerging evidence suggests that liquid biopsy approaches, particularly the analysis of circulating tumor DNA, show promise as prognostic biomarkers in acute myeloid leukemia. Initial studies indicate that ctDNA monitoring may provide prognostic information comparable to traditional bone marrow-based assessments in specific clinical contexts, though validation studies and standardized protocols are still needed for broader clinical implementation (68, 69).

Exosome analysis represents a promising research direction for future MRD monitoring, with theoretical advantages including non-invasive sampling and potential for frequent monitoring (70). However, exosome-based MRD approaches remain investigational and require standardization of isolation methods, validation of biomarker panels, and clinical trials before implementation. The 2021 ELN MRD consensus guidelines focus on established technologies and do not currently include pathways for exosome-based monitoring (17).

### 4.4 Clinical decision integration

European hematology guidelines increasingly emphasize personalized treatment approaches based on validated genetic and molecular biomarkers for risk stratification. While exosome research shows promise in preclinical and early clinical studies, these approaches remain investigational and are not currently incorporated into clinical decision algorithms or European treatment guidelines. Further validation studies and regulatory approval would be required before exosome biomarkers could be integrated into standard clinical practice (71). While exosomes show promise as biomarkers and therapeutic agents, current research indicates that specific clinical trials correlating baseline exosomal signatures with treatment selection for targeted therapies, immunotherapies, and conventional chemotherapy remain largely in preclinical or early research phases. Although exosome-based approaches are being investigated for cancer diagnosis and therapy, clinical validation of treatment selection strategies requires further development (48, 72, 73).

While individual exosome-based diagnostic tests like the ExoDx Prostate Test have reached clinical implementation, the development of comprehensive clinical decision support tools that integrate exosome analysis with conventional diagnostic markers remains largely conceptual (72). Current challenges include a lack of standardization in exosome isolation and analysis, limited integration capabilities with existing clinical workflows, and the need for further validation studies. Most clinical decision support tools in current use focus on established biomarkers and risk calculators rather than incorporating emerging technologies like exosome analysis (74).

### 4.5 Regulatory validation and clinical trial integration

Current clinical validation efforts focus on demonstrating the clinical utility of exosome biomarkers within regulatory frameworks established by the EMA and FDA. Two exosome-based diagnostics have received FDA regulatory designations: the ExoDx Prostate IntelliScore test received Breakthrough Device Designation in 2019, and Guardant360CDx received full FDA approval in 2020 as the first liquid biopsy companion diagnostic using next-generation sequencing technology (72, 75). However, there are currently no FDA-approved exosome therapeutic products, as exosome products are regulated as drugs and biological products under the Public Health Service Act Section 351 and require premarket review and approval (11). The regulatory strategy for exosome diagnostics involves demonstration of analytical validity, clinical validity, and clinical utility through appropriately designed studies that meet regulatory standards for biomarker qualification.

Clinical trials are underway to validate the efficacy of exosomal biomarkers for enhancing diagnostic accuracy and predicting treatment responses, though challenges such as the lack of efficient isolation and analysis methods and the need for validated biomarkers in clinical settings remain (76). In clinical trials, exosomes are being used as biomarkers, cell-free therapy, drug delivery systems, and cancer vaccines, with biomarker applications representing approximately 50% of registered studies (77). While their role as biomarkers for early detection and patient monitoring is promising, scalability and regulatory challenges remain, and exosomes and their usage need to comply with good manufacturing practice (GMP), with both the European Medicines Agency and US Food and Drug Administration releasing recommendations for advanced therapy production (72, 76, 77).

### 4.6 Future integration with digital health

Machine learning algorithms demonstrate transformative potential for addressing fundamental clinical challenges in exosomal biomarker implementation (78). Random forest and ensemble models achieve superior discrimination of malignant from benign exosomal profiles, with recent studies demonstrating AUROC scores exceeding 0.91 for pan-cancer detection across plasma, serum, and urine-derived exosomes compared to single-biomarker approaches that typically achieve sensitivities below 75% (79). Surface-enhanced Raman spectroscopy combined with artificial intelligence has achieved 90.2% sensitivity and 94.4% specificity for early-stage cancer detection across six tumor types, representing significant improvements over conventional diagnostic approaches. These AI-driven models excel at integrating heterogeneous exosomal data encompassing protein signatures, miRNA panels, and surface marker profiles to identify subtle pattern differences that escape traditional analytical methods (80). Cost reduction represents a critical advantage through algorithmic optimization of biomarker panels, where machine learning can identify minimal combinations of markers needed for accurate diagnosis while reducing assay complexity and processing requirements (81). Furthermore, AI algorithms can compensate for inter-laboratory variability by incorporating correction factors during model training, directly addressing standardization challenges that currently impede clinical translation. SERS biosensors integrated with machine learning demonstrate clinical feasibility for automated exosome classification, with FlowSOM algorithms now enabling proteomic-based clustering that enhances diagnostic specificity while reducing manual interpretation requirements (82).

Predictive modeling for treatment response represents the most clinically impactful application of AI in exosomal biomarker analysis (83). Deep learning frameworks demonstrate remarkable accuracy in predicting therapeutic responses, with recent studies achieving over 80% accuracy in identifying cancer patients likely to respond to specific chemotherapeutic regimens based on exosomal molecular signatures (84). Machine learning algorithms can integrate exosomal miRNA panels, protein signatures, and clinical variables to generate personalized treatment recommendations, particularly valuable for identifying second-line therapies in patients failing standard-of-care treatments (85). These predictive models address the fundamental challenge of treatment selection in hematological malignancies, where heterogeneous disease presentation and acquired resistance mechanisms complicate therapeutic decision-making. The ability to predict treatment failure before clinical manifestation enables proactive therapy modifications that can significantly improve patient outcomes while reducing unnecessary drug exposure and associated toxicities.

Standardization challenges that have historically impeded exosome clinical translation are increasingly addressable through AI-driven harmonization approaches (86). Machine learning algorithms can compensate for inter-laboratory variability by identifying and correcting systematic biases in isolation protocols, analytical methods, and measurement platforms across different healthcare institutions (87). Advanced harmonization frameworks leverage artificial intelligence to standardize unstructured clinical data, integrate diverse analytical platforms, and establish quality control metrics that ensure reproducible results regardless of the laboratory environment (88). These AI-enabled standardization approaches are essential for multi-center clinical trials and regulatory approval pathways, as they provide the analytical consistency required for validating exosomal biomarkers across diverse patient populations and clinical settings.

Regulatory frameworks are rapidly evolving to accommodate AI-enhanced exosomal diagnostics, with the FDA recently issuing draft guidance specifically addressing artificial intelligence applications in drug and biological product development (89). The integration of AI algorithms with exosomal biomarker platforms creates sophisticated clinical decision support systems that can provide real-time treatment recommendations while maintaining the rigorous validation standards required for regulatory approval (90). These systems must demonstrate analytical validity, clinical validity, and clinical utility through appropriately designed studies that meet regulatory standards for biomarker qualification, with particular attention to algorithm transparency and risk management protocols (91). While the European Health Data Space aims to enable citizens to gain secure access to their electronic health data and establish interoperability requirements (92), the integration of AI-powered exosomal analysis with existing clinical decision support systems requires comprehensive validation and standardized implementation protocols that can accommodate the high-throughput requirements of routine clinical practice (78, 79, 93).

### 4.7 Clinical trial landscape and development pipeline

Despite extensive preclinical research, the clinical trial landscape for exosomal biomarkers in hematological malignancies remains limited. Current registered trials focus primarily on exosome characterization (NCT03275363, NCT03944603) (94) rather than biomarker validation for hematological disorders. Clinical trials evaluating exosome-based immunotherapy (NCT05375604) target solid tumors, with limited representation of hematological malignancies (95).

The development pipeline indicates that most promising biomarkers require progression through the following validated clinical development stages: (1) Analytical Validation: Standardized isolation and detection protocols across laboratories, (2) Clinical Validation: Multi-center studies with adequate sample sizes (n>200) and appropriate controls, (3) Clinical Utility: Integration with existing diagnostic algorithms and demonstration of clinical decision impact, and (4) Regulatory Approval: Meeting FDA/EMA requirements for analytical validity, clinical validity, and clinical utility as defined by established biomarker qualification frameworks.

### 4.8 Leukemia heterogeneity and clinical implementation challenges

The precise treatment of acute myeloid leukemia is impeded by the disease’s aggressive and heterogeneous nature, characterized by genetic abnormalities, extensive epigenetic changes, and abnormal tumor microenvironment (16). Genetic and epigenetic variations within tumor cells lead to diverse treatment responses, making it difficult to predict therapeutic outcomes from exosomal biomarker panels (61). Single-cell chronic myeloid leukemia cell line models may not fully capture the complexity and heterogeneity of disease *in vivo*, necessitating validation approaches that account for patient-to-patient molecular variability (27).

Most tumors exhibit heterogeneity comprising different cell types with diverse molecular profiles, and exosomes released by various tumor-resident cells provide a more comprehensive view of tumor heterogeneity than single tissue biopsies. However, this heterogeneity poses analytical challenges, as bulk-level analysis methods like mass spectroscopy may give inaccurate results in detecting exosome heterogeneity (82). The heterogeneity of exosome composition and the lack of standardized protocols for isolation, characterization, and modification challenge reproducibility and quality control across diverse patient populations (96).

## 5 Therapeutic applications and clinical translation

### 5.1 Exosome-based drug delivery and therapeutic interventions

Clinical trials are evaluating engineered exosomes as drug delivery vehicles, showing enhanced biocompatibility compared to synthetic nanoparticles. European initiatives include exosomes loaded with chemotherapeutic agents and siRNA, though standardization challenges remain (16, 97).

Direct intervention strategies targeting exosome-mediated resistance mechanisms show promise in clinical trials. Extracorporeal hemofiltration for selective removal of tumor-derived exosomes has demonstrated feasibility in Phase I trials, with preliminary evidence suggesting improved treatment responses when combined with conventional chemotherapy (98). The heparanase inhibitor Roneparstat demonstrated clinical feasibility in multiple myeloma patients through the NCT01764880 trial, providing proof-of-concept for targeting exosome-mediated communication networks in hematological malignancies (99).

### 5.2 Immunomodulatory and combination approaches

Exosome-based immunotherapies demonstrate particular promise in hematological malignancies, with completed clinical trials of dendritic cell-derived exosomes showing safety and immunogenicity in melanoma and lung cancer patients (100), while preclinical studies demonstrate enhanced antileukemic immunity when dendritic cells are pulsed with leukemia cell-derived exosomes (101). CAR-T cell-derived exosomes represent an innovative approach combining targeting specificity with reduced cytotoxicity compared to cellular counterparts, currently advancing through preclinical validation with demonstrated safety advantages (55).

The European LeukemiaNet collaborative framework provides an established infrastructure for multi-center evaluation across diverse patient populations through 220 participating centers in 44 countries (102), though specific clinical trials evaluating combination approaches targeting both primary leukemic cells and their exosome-mediated support networks remain to be initiated. Current evidence supports the therapeutic potential of exosome-based approaches, with preliminary preclinical data suggesting enhanced efficacy compared to single-agent approaches.

## 6 Implementation challenges and regulatory pathways

### 6.1 Regulatory framework

The regulatory landscape for exosome therapeutics is complex and varies across countries due to their unique intracellular mechanisms of action, with the diversity of manufacturing techniques rendering standardization challenging and leading to a fragmented regulatory landscape (11). Currently, no FDA-approved exosome products exist, and as of October 2023, the FDA has issued six warning letters regarding exosome products that failed to meet regulatory requirements (103).

These products are regulated as drugs, devices, and biological products under the Federal Food, Drug, and Cosmetic Act and the Public Health Service Act, requiring premarket review and approval. The EMA framework for advanced therapy medicinal products provides regulatory guidance, with exosomes containing functional transgenic mRNAs classified as gene therapy medicinal products requiring specialized oversight that addresses manufacturing consistency, batch-to-batch variability, and long-term stability (104).

Regulatory bodies are developing comprehensive guidelines addressing characterization, safety, and efficacy of these products, which is vital for mitigating concerns related to immunogenicity and long-term effects. Challenges remain in managing exosome source variability, scaling up production, standardizing isolation and characterization protocols, and ensuring batch-to-batch consistency regarding safety and efficacy. The timeline for regulatory approval typically extends 8–15 years from preclinical development through market authorization, with cost estimates for meeting reasonable manufacturing practice requirements ranging from $10–50 million for facility establishment and validation (11, 104, 105).

### 6.2 Standardization and healthcare integration

The lack of standardized protocols for exosome isolation, characterization, and analysis represents a fundamental implementation challenge. International standardization efforts, particularly through ISEV’s MISEV2023 guidelines, are addressing pre-analytical variables and analytical procedures; however, standardization remains challenging due to the diverse manufacturing techniques and fragmented regulatory landscapes (11).

The integration of advanced therapy medicinal products, including exosome therapeutics, into the healthcare system requires consideration of economic, logistical, and educational factors that influence adoption. Cost-effectiveness analyses must address the temporal misalignment between high upfront costs and long-term benefits, particularly within resource-constrained environments. ATMP management requires specialized centers of excellence with appropriate funding, equipment, and healthcare professional expertise to ensure effective patient access (106).

Healthcare integration faces additional implementation barriers beyond standardization challenges. Current exosome isolation methods suffer from operation complexity, time consumption, large sample volumes, and low purity, posing significant challenges for downstream analysis in clinical settings (107). Training requirements for laboratory personnel represent a substantial barrier, as exosome analysis requires specialized competencies in vesicle biology, advanced analytical techniques, and quality control procedures that differ significantly from conventional clinical laboratory workflows. The absence of reference materials and validated control samples further complicates the implementation of standardized protocols across diverse healthcare settings.

### 6.3 Training and education requirements

Successful implementation requires comprehensive training programs that address both technical competencies and clinical interpretation skills. Research-focused educational initiatives in extracellular vesicle science are emerging across Europe through international collaborations and professional societies. MISEV2023 guidelines recommend that researchers actively promote standardized approaches during laboratory meetings, journal clubs, seminars, workshops, and conferences to ensure proper training in EV methodologies. European mobility programs facilitate knowledge exchange and skill development among early-career researchers in the extracellular vesicle field (108).

## 7 Future perspectives and clinical translation

### 7.1 Emerging technologies and precision medicine integration

Future exosome applications are being shaped by advancing analytical technologies, which provide enhanced sensitivity, specificity, and throughput suitable for clinical implementation. Single extracellular vesicle analysis technologies are providing unprecedented resolution for disease characterization, while point-of-care testing platforms are being developed to democratize access across healthcare settings through integrated microfluidic biosensors and portable diagnostic systems (109, 110). Integration of artificial intelligence and machine learning approaches offers opportunities for identifying complex biomarker patterns and optimizing diagnostic accuracy (79).

The alignment of exosome research with precision medicine initiatives positions these technologies as emerging components of personalized healthcare approaches. Integration of exosomes with pharmacogenomics and other multi-omics approaches offers potential opportunities for enhanced molecular profiling and treatment optimization, though clinical implementation remains largely in development (111).

### 7.2 Accessibility and research priorities

The clinical translation of exosome diagnostics faces specific implementation challenges that require targeted research. Priority areas include: (1) validation of standardized isolation and characterization protocols across laboratories, (2) establishment of reference materials and quality control measures, (3) health economic evaluations comparing exosome-based liquid biopsies to tissue biopsy costs and outcomes, and (4) regulatory science studies supporting approval pathways.

Multi-center validation studies should evaluate diagnostic performance across diverse populations, with particular attention to analytical validity, clinical validity, and clinical utility as defined by established frameworks. Research networks capable of harmonizing protocols and sharing biobank resources will be essential for generating the evidence base required for regulatory approval and health technology assessment.

## 8 Comparative analysis with alternative liquid biopsy approaches

The clinical implementation of exosomal biomarkers must be evaluated within the context of competing liquid biopsy technologies to understand their relative advantages and limitations. Methylome-based approaches represent the most directly comparable alternative, offering complementary mechanisms for non-invasive cancer detection through analysis of circulating cell-free DNA (cfDNA) methylation patterns.

Recent studies demonstrate that methylome-based liquid biopsies achieve superior diagnostic performance in hematological malignancies, with cfMeDIP-seq technology discriminating acute myeloid leukemia from healthy individuals with area under the curve values of 0.98 (112). The cell-free methylated DNA immunoprecipitation approach provides enhanced sensitivity compared to mutation-based detection because aberrant methylation is both more prevalent and pervasive than genetic mutations, while maintaining tissue specificity that enables tumor origin determination (113). These methylation-based platforms demonstrate established analytical validity with standardized protocols that integrate seamlessly into existing laboratory workflows, requiring minimal specialized equipment beyond standard DNA extraction and PCR capabilities.

In contrast, exosomal biomarkers face substantial standardization challenges that currently limit clinical translation. Ultracentrifugation, considered the gold standard for exosome isolation, recovers only 5-25% of total exosomes while requiring specialized equipment and extended processing times that compromise scalability for routine clinical use (114). Commercial isolation kits address some technical barriers but remain expensive and unsuitable for high-throughput processing, creating cost-effectiveness challenges for widespread implementation. The absence of standardized protocols for exosome isolation, characterization, and analysis represents a fundamental barrier to reproducibility across laboratories, contrasting sharply with the established methodological consensus surrounding cfDNA methylation analysis.

Despite these implementation challenges, exosomal biomarkers offer unique biological advantages through their comprehensive molecular cargo encompassing RNA, proteins, and lipids that reflect real-time cellular communication networks. This multimodal information content provides functional insights into disease mechanisms that methylome-based approaches cannot capture, particularly regarding intercellular signaling pathways that drive treatment resistance and disease progression. The protective vesicular membrane structure maintains RNA stability under conditions that degrade free circulating nucleic acids, enabling detection of transcriptomic signatures that complement genomic and epigenomic analyses (115).

Cost-effectiveness analyses favor methylome-based approaches for near-term clinical implementation. Digital PCR-based methylation assays require minimal sample volumes and provide rapid turnaround times compatible with clinical decision-making timelines. At the same time, established DNA-based workflows reduce training requirements and infrastructure investments (116). Conversely, exosome isolation typically yields less than 1 μg protein per milliliter of culture medium, necessitating larger sample volumes and more complex processing protocols that increase both direct costs and technical complexity.

The regulatory landscape further distinguishes these approaches, with methylome-based diagnostics demonstrating clearer pathways toward clinical approval through established precedents in cfDNA analysis. Two methylation-based liquid biopsy tests have received FDA breakthrough device designations, while no exosomal biomarkers have achieved regulatory approval for hematological malignancies, reflecting the maturation gap between these technological approaches (117).

However, recent evidence suggests that combining exosomal and cfDNA analyses may provide synergistic diagnostic performance superior to either approach alone. Studies demonstrate that integrating exosomal RNA profiling with cfDNA methylation analysis enhances sensitivity and specificity compared to single-modality approaches, suggesting complementary rather than competitive clinical applications (118). This integration strategy addresses the primary limitation of methylome-based approaches, which focus exclusively on epigenetic information while missing post-transcriptional regulatory mechanisms captured by exosomal RNA cargo.

The differential advantages position these technologies for distinct clinical niches within precision leukemia medicine. Methylome-based approaches demonstrate superior feasibility for population screening and routine monitoring applications where standardization, cost-effectiveness, and regulatory approval represent primary considerations. Exosomal biomarkers may find optimal utility in specialized applications where their unique biological information provides clinical advantages that justify increased complexity and cost, particularly in treatment resistance prediction and personalized therapy selection, where functional cellular communication networks drive clinical outcomes.

Future clinical practice will likely benefit from integrated liquid biopsy platforms that leverage the strengths of multiple biomarker types rather than viewing them as competing technologies, with methylome-based approaches providing the standardized foundation for routine clinical implementation. At the same time, exosomal analyses contribute specialized functional insights for precision medicine applications.

## 9 Conclusion

Exosomes offer genuine potential for advancing precision medicine in leukemia, particularly for diagnostic and monitoring applications. However, the translation from promising research to routine clinical practice faces substantial challenges that require realistic expectations and sustained collaborative effort.

The most significant barriers remain the standardization of isolation and analytical methods, comprehensive clinical validation, and navigating complex regulatory pathways for advanced therapy medicinal products. While individual studies demonstrate encouraging diagnostic performance, the field lacks the multi-center validation studies and consensus protocols necessary for widespread clinical implementation. Therapeutic applications, though scientifically compelling, remain largely in early development phases.

While this review highlights the significant potential of exosomal biomarkers in leukemia, it is essential to acknowledge the current clinical development reality. The vast majority of promising biomarkers remain in preclinical or early validation phases, with substantial gaps between laboratory discoveries and clinical implementation.

Progress will likely be incremental, with specialized diagnostic applications potentially reaching clinical utility before broader therapeutic implementations. Success will depend on focused efforts in standardization, rigorous validation studies, and realistic timelines that prioritize quality and reproducibility over speed of translation. The promise of exosomes in leukemia management is real, but its realization requires continued investment in fundamental methodological challenges alongside clinical development.

## References

[1] Hushmandi K Saadat SH Raei M Aref AR Reiter RJ Nabavi N. The science of exosomes: understanding their formation, capture, and role in cellular communication. Pathology-Research Practice. (2024) 155388. doi: 10.1016/j.prp.2024.155388, PMID: 38850846

[2] Martins B Pires M Ambrósio AF Girão H Fernandes R. Contribution of extracellular vesicles for the pathogenesis of retinal diseases: Shedding light on blood-retinal barrier dysfunction. J Biomed Science. (2024) 31:48. doi: 10.1186/s12929-024-01036-3, PMID: 38730462 PMC11088087

[3] Allegra A Petrarca C Di Gioacchino M Casciaro M Musolino C Gangemi S. Exosome-mediated therapeutic strategies for management of solid and hematological Malignancies. Cells. (2022) 11:1128. doi: 10.3390/cells11071128, PMID: 35406692 PMC8997895

[4] Cariello M Squilla A Piacente M Venutolo G Fasano A. Drug resistance: the role of exosomal miRNA in the microenvironment of hematopoietic tumors. Molecules. (2022) 28:116. doi: 10.3390/molecules28010116, PMID: 36615316 PMC9821808

[5] Wang X Tian L Lu J Ng IO-L. Exosomes and cancer-Diagnostic and prognostic biomarkers and therapeutic vehicle. Oncogenesis. (2022) 11:54. doi: 10.1038/s41389-022-00431-5, PMID: 36109501 PMC9477829

[6] Li J Zhang Y Dong P-Y Yang G-M Gurunathan S. A comprehensive review on the composition, biogenesis, purification, and multifunctional role of exosome as delivery vehicles for cancer therapy. Biomedicine Pharmacotherapy. (2023) 165:115087. doi: 10.1016/j.biopha.2023.115087, PMID: 37392659

[7] Wang J Yue B-L Huang Y-Z Lan X-Y Liu W-J Chen H. Exosomal RNAs: novel potential biomarkers for diseases—a review. Int J Mol Sci. (2022) 23:2461. doi: 10.3390/ijms23052461, PMID: 35269604 PMC8910301

[8] Trino S Lamorte D Caivano A De Luca L Sgambato A Laurenzana I. Clinical relevance of extracellular vesicles in hematological neoplasms: from liquid biopsy to cell biopsy. Leukemia. (2021) 35:661–78. doi: 10.1038/s41375-020-01104-1, PMID: 33299143 PMC7932927

[9] Escudier B Dorval T Chaput N André F Caby M-P Novault S. Vaccination of metastatic melanoma patients with autologous dendritic cell (DC) derived-exosomes: results of the first phase I clinical trial. J Trans Med. (2005) 3:1–13. doi: 10.1186/1479-5876-3-10, PMID: 15740633 PMC554765

[10] Perocheau D Touramanidou L Gurung S Gissen P Baruteau J. Clinical applications for exosomes: Are we there yet? Br J Pharmacol. (2021) 178:2375–92. doi: 10.1111/bph.15432, PMID: 33751579 PMC8432553

[11] Wang CK Tsai TH Lee CH. Regulation of exosomes as biologic medicines: Regulatory challenges faced in exosome development and manufacturing processes. Clin Trans Science. (2024) 17:e13904. doi: 10.1111/cts.13904, PMID: 39115257 PMC11307316

[12] Schuessler-Lenz M Herberts C Reischl I Ruiz S Celis P Beuneu C. Marketing regulatory oversight of advanced therapy medicinal products in Europe. In: Regulatory Aspects of Gene Therapy and Cell Therapy Products: A Global Perspective (2023) Springer, Cham. p. 1–21. 10.1007/978-3-031-34567-8_1 37526839

[13] Forte D Barone M Palandri F Catani L. The “vesicular intelligence” strategy of blood cancers. Genes. (2021) 12:416. doi: 10.3390/genes12030416, PMID: 33805807 PMC7999060

[14] Sobas M Elicegui JM Ramiro AV González T Hernandez-Sanchez A Melchor RA. Harmony alliance provides a machine learning researching tool to predict the risk of relapse after first remission in AML patients treated without allogeneic hematopoietic stem cell transplantation. Blood. (2021) 138:4041. doi: 10.1182/blood-2021-149521

[15] Karabay AZ Ozkan T Karadag Gurel A Koc A Hekmatshoar Y Sunguroglu A. Identification of exosomal microRNAs and related hub genes associated with imatinib resistance in chronic myeloid leukemia. Naunyn-Schmiedeberg’s Arch Pharmacol. (2024) 397:9701–21. doi: 10.1007/s00210-024-03198-1, PMID: 38916832 PMC11582232

[16] Wang W Wu X Zheng J Yin R Li Y Wu X. Utilizing exosomes as sparking clinical biomarkers and therapeutic response in acute myeloid leukemia. Front Immunol. (2024) 14:1315453. doi: 10.3389/fimmu.2023.1315453, PMID: 38292478 PMC10824954

[17] Heuser M Freeman SD Ossenkoppele GJ Buccisano F Hourigan CS Ngai LL. 2021 Update on MRD in acute myeloid leukemia: a consensus document from the European LeukemiaNet MRD Working Party. Blood J Am Soc Hematol. (2021) 138:2753–67. doi: 10.1182/blood.2021013626, PMID: 34724563 PMC8718623

[18] Oksvold MP Kullmann A Forfang L Kierulf B Li M Brech A. Expression of B-cell surface antigens in subpopulations of exosomes released from B-cell lymphoma cells. Clin Ther. (2014) 36:847–862.e1. doi: 10.1016/j.clinthera.2014.05.010, PMID: 24952935

[19] Landberg N von Palffy S Askmyr M Lilljebjörn H Sandén C Rissler M. CD36 defines primitive chronic myeloid leukemia cells less responsive to imatinib but vulnerable to antibody-based therapeutic targeting. Hematologica. (2017) 103:447. doi: 10.3324/haematol.2017.169946, PMID: 29284680 PMC5830390

[20] Gonzalez LL Garrie K Turner MD. Role of S100 proteins in health and disease. Biochim Biophys Acta (BBA)-Molecular Cell Res. (2020) 1867:118677. doi: 10.1016/j.bbamcr.2020.118677, PMID: 32057918

[21] Gil-Kulik P Kluz N Przywara D Petniak A Wasilewska M Frączek-Chudzik N. Potential use of exosomal non-coding microRNAs in leukemia therapy: A systematic review. Cancers. (2024) 16:3948. doi: 10.3390/cancers16233948, PMID: 39682135 PMC11639955

[22] Kang K-W Gim J-A Hong S Kim HK Choi Y J-h P. Use of extracellular vesicle microRNA profiles in patients with acute myeloid leukemia for the identification of novel biomarkers. PloS One. (2024) 19:e0306962. doi: 10.1371/journal.pone.0306962, PMID: 39178208 PMC11343415

[23] Li C Zhou T Chen J Li R Chen H Luo S. The role of Exosomal miRNAs in cancer. J Trans Med. (2022) 20:1–15. doi: 10.1186/s12967-021-03215-4, PMID: 34980158 PMC8722109

[24] Hornick NI Huan J Doron B Goloviznina NA Lapidus J Chang BH. Serum exosome microRNA as a minimally-invasive early biomarker of AML. Sci Rep. (2015) 5:11295. doi: 10.1038/srep11295, PMID: 26067326 PMC4650871

[25] Calin GA Cimmino A Fabbri M Ferracin M Wojcik SE Shimizu M. MiR-15a and miR-16–1 cluster functions in human leukemia. Proc Natl Acad Sci. (2008) 105:5166–71. doi: 10.1073/pnas.0800121105, PMID: 18362358 PMC2278188

[26] Hrdinova T Toman O Dresler J Klimentova J Salovska B Pajer P. Exosomes released by imatinib-resistant K562 cells contain specific membrane markers, IFITM3, CD146 and CD36 and increase the survival of imatinib-sensitive cells in the presence of imatinib. Int J Oncol. (2020) 58:238–50. doi: 10.3892/ijo.2020.5163, PMID: 33491750

[27] Navakanitworakul R Saelue P Penglong T Molika P Nokchan N Tansila N. Exosomal miRNA expression profiling in patients with imatinib resistant Chronic myeloid leukemia: A pilot study. PloS One. (2025) 20:e0331479. doi: 10.1371/journal.pone.0331479, PMID: 40880391 PMC12396705

[28] Damanti CC Gaffo E Lovisa F Garbin A Di Battista P Gallingani I. MiR-26a-5p as a reference to normalize microRNA qRT-PCR levels in plasma exosomes of pediatric hematological Malignancies. Cells. (2021) 10:101. doi: 10.3390/cells10010101, PMID: 33429910 PMC7827902

[29] Falini B. NPM1-mutated acute myeloid leukemia: new pathogenetic and therapeutic insights and open questions. Am J hematology. (2023) 98:1452–64. doi: 10.1002/ajh.26989, PMID: 37317978

[30] Umezu T Tadokoro H Azuma K Yoshizawa S Ohyashiki K Ohyashiki JH. Exosomal miR-135b shed from hypoxic multiple myeloma cells enhances angiogenesis by targeting factor-inhibiting HIF-1. Blood J Am Soc Hematology. (2014) 124:3748–57. doi: 10.1182/blood-2014-05-576116, PMID: 25320245 PMC4263983

[31] Kang K-W Jung J-H Hur W Park J Shin H Choi B. The potential of exosomes derived from chronic myelogenous leukemia cells as a biomarker. Anticancer Res. (2018) 38:3935–42. doi: 10.21873/anticanres.12679, PMID: 29970515

[32] Dilsiz N. A comprehensive review on recent advances in exosome isolation and characterization: Toward clinical applications. Trans Oncol. (2024) 50:102121. doi: 10.1016/j.tranon.2024.102121, PMID: 39278189 PMC11418158

[33] Lener T Gimona M Aigner L Börger V Buzas E Camussi G. Applying extracellular vesicles based therapeutics in clinical trials–an ISEV position paper. J extracellular vesicles. (2015) 4:30087. doi: 10.3402/jev.v4.30087, PMID: 26725829 PMC4698466

[34] Coumans FA Brisson AR Buzas EI Dignat-George F Drees EE El-Andaloussi S. Methodological guidelines to study extracellular vesicles. Circ Res. (2017) 120:1632–48. doi: 10.1161/CIRCRESAHA.117.309417, PMID: 28495994

[35] Cheng K Kalluri R. Guidelines for clinical translation and commercialization of extracellular vesicles and exosomes based therapeutics. Extracellular Vesicle. (2023) 2:100029. doi: 10.1016/j.vesic.2023.100029

[36] Ramnauth N Neubarth E Makler-Disatham A Sher M Soini S Merk V. Development of a microfluidic device for exosome isolation in point-of-care settings. Sensors. (2023) 23:8292. doi: 10.3390/s23198292, PMID: 37837121 PMC10574868

[37] Zhang X Jia L Liu N Zhao Y Zhang T Xie X. Inside-out extracellular vesicles-like biomimetic magnetic nanoparticles for efficient screening P-Glycoprotein inhibitors to overcome cancer multidrug resistance. Colloids Surfaces B: Biointerfaces. (2023) 222:113134. doi: 10.1016/j.colsurfb.2023.113134, PMID: 36630772

[38] Ludwig N Whiteside TL Reichert TE. Challenges in exosome isolation and analysis in health and disease. Int J Mol Sci. (2019) 20:4684. doi: 10.3390/ijms20194684, PMID: 31546622 PMC6801453

[39] Zhang F Burghardt T Höhn M Wagner E. Dual effect by chemical electron transfer enhanced siRNA lipid nanoparticles: reactive oxygen species-triggered tumor cell killing aggravated by Nrf2 gene silencing. Pharmaceutics. (2024) 16:779. doi: 10.3390/pharmaceutics16060779, PMID: 38931900 PMC11207527

[40] Coughlan C Bruce KD Burgy O Boyd TD Michel CR Garcia-Perez JE. Exosome isolation by ultracentrifugation and precipitation and techniques for downstream analyses. Curr Protoc Cell Biol. (2020) 88:e110. doi: 10.1002/cpcb.110, PMID: 32633898 PMC8088761

[41] Welsh JA Goberdhan DC O’Driscoll L Buzas EI Blenkiron C Bussolati B. Minimal information for studies of extracellular vesicles (MISEV2023): From basic to advanced approaches. J extracellular vesicles. (2024) 13:e12404. doi: 10.1002/jev2.12404, PMID: 38326288 PMC10850029

[42] Chen C Zong S Liu Y Wang Z Zhang Y Chen B. Profiling of exosomal biomarkers for accurate cancer identification: Combining DNA-PAINT with machine-learning-based classification. Small. (2019) 15:1901014. doi: 10.1002/smll.201901014, PMID: 31478613

[43] Wang F Wang C Chen S Wei C Ji J Liu Y. Identification of blood-derived exosomal tumor RNA signatures as noninvasive diagnostic biomarkers for multi-cancer: a multi-phase, multi-center study. Mol Cancer. (2025) 24:60. doi: 10.1186/s12943-025-02271-4, PMID: 40025576 PMC11871737

[44] Kumar B Garcia M Weng L Jung X Murakami J Hu X. Acute myeloid leukemia transforms the bone marrow niche into a leukemia-permissive microenvironment through exosome secretion. Leukemia. (2018) 32:575–87. doi: 10.1038/leu.2017.259, PMID: 28816238 PMC5843902

[45] Mineo M Garfield SH Taverna S Flugy A De Leo G Alessandro R. Exosomes released by K562 chronic myeloid leukemia cells promote angiogenesis in a Src-dependent fashion. Angiogenesis. (2012) 15:33–45. doi: 10.1007/s10456-011-9241-1, PMID: 22203239 PMC3595015

[46] Chen S Chen X Luo Q Liu X Wang X Cui Z. Retinoblastoma cell-derived exosomes promote angiogenesis of human vesicle endothelial cells through microRNA-92a-3p. Cell Death Disease. (2021) 12:695. doi: 10.1038/s41419-021-03986-0, PMID: 34257272 PMC8277798

[47] Ahmadi M Rezaie J. Tumor cells derived-exosomes as angiogenenic agents: possible therapeutic implications. J Trans Med. (2020) 18:249. doi: 10.1186/s12967-020-02426-5, PMID: 32571337 PMC7310379

[48] Li J Wang J Chen Z. Emerging role of exosomes in cancer therapy: progress and challenges. Mol Cancer. (2025) 24:13. doi: 10.1186/s12943-024-02215-4, PMID: 39806451 PMC11727182

[49] Zhao Z Shuang T Gao Y Lu F Zhang J He W. Targeted delivery of exosomal miR-484 reprograms tumor vasculature for chemotherapy sensitization. Cancer Letters. (2022) 530:45–58. doi: 10.1016/j.canlet.2022.01.011, PMID: 35051533

[50] Xu Z Zeng S Gong Z Yan Y. Exosome-based immunotherapy: a promising approach for cancer treatment. Mol cancer. (2020) 19:1–16. doi: 10.1186/s12943-020-01278-3, PMID: 33183286 PMC7661275

[51] Marleau AM Chen C-S Joyce JA Tullis RH. Exosome removal as a therapeutic adjuvant in cancer. J Trans Med. (2012) 10:1–12. doi: 10.1186/1479-5876-10-134, PMID: 22738135 PMC3441244

[52] Poggio M Hu T Pai C-C Chu B Belair CD Chang A. Suppression of exosomal PD-L1 induces systemic anti-tumor immunity and memory. Cell. (2019) 177:414–427.e13. doi: 10.1016/j.cell.2019.02.016, PMID: 30951669 PMC6499401

[53] Galli M Chatterjee M Grasso M Specchia G Magen H Einsele H. Phase I study of the heparanase inhibitor roneparstat: An innovative approach for multiple myeloma therapy. Hematologica. (2018) 103:e469. doi: 10.3324/haematol.2017.182865, PMID: 29700168 PMC6165822

[54] Wang D Zhou F He L Wang X Song L Wang H. AML cell-derived exosomes suppress the activation and cytotoxicity of NK cells in AML via PD-1/PD-L1 pathway. Cell Biol Int. (2024) 48:1588–98. doi: 10.1002/cbin.12225, PMID: 39030886

[55] Fu W Lei C Liu S Cui Y Wang C Qian K. CAR exosomes derived from effector CAR-T cells have potent antitumor effects and low toxicity. Nat Commun. (2019) 10:4355. doi: 10.1038/s41467-019-12321-3, PMID: 31554797 PMC6761190

[56] Zhang H Xia J Wang X Wang Y Chen J He L. Recent progress of exosomes in hematological Malignancies: pathogenesis, diagnosis, and therapeutic strategies. Int J Nanomedicine. (2024) 19:11611–31. doi: 10.2147/IJN.S479697, PMID: 39539968 PMC11559222

[57] Castelo-Branco L Pellat A Martins-Branco D Valachis A Derksen J Suijkerbuijk K. ESMO guidance for reporting oncology real-world evidence (GROW). Ann Oncol. (2023) 34:1097–112. doi: 10.1016/j.annonc.2023.10.001, PMID: 37848160

[58] Moloudizargari M Hekmatirad S Mofarahe ZS Asghari MH. Exosomal microRNA panels as biomarkers for hematological Malignancies. Curr Problems Cancer. (2021) 45:100726. doi: 10.1016/j.currproblcancer.2021.100726, PMID: 33752898

[59] Allegra A Alonci A Penna G Innao V Gerace D Rotondo F. The cancer stem cell hypothesis: a guide to potential molecular targets. Cancer Invest. (2014) 32:470–95. doi: 10.3109/07357907.2014.958231, PMID: 25254602

[60] Neaga A Bagacean C Tempescul A Jimbu L Mesaros O Blag C. MicroRNAs associated with a good prognosis of acute myeloid leukemia and their effect on macrophage polarization. Front Immunol. (2021) 11:582915. doi: 10.3389/fimmu.2020.582915, PMID: 33519805 PMC7845488

[61] Rahgoshay M Atashi A Vaezi M Ajorloo M Amini-Kafiabad S Ahmadvand M. Engineered exosomes: advanced nanocarriers for targeted therapy and drug delivery in hematological Malignancies. Cancer Nanotechnology. (2025) 16:33. doi: 10.1186/s12645-025-00334-1

[62] Guo H-Z Feng R-X Zhang Y-J Yu Y-H Lu W Liu J-J. A CD36-dependent non-canonical lipid metabolism program promotes immune escape and resistance to hypomethylating agent therapy in AML. Cell Rep Med. (2024) 5:101592. doi: 10.1016/j.xcrm.2024.101592, PMID: 38843841 PMC11228649

[63] Rajapaksa US Jin C Dong T. Malignancy and IFITM3: friend or foe? Front Oncol. (2020) 10:593245. doi: 10.3389/fonc.2020.593245, PMID: 33364194 PMC7753217

[64] Fathi AT Chabner BA. FLT3 inhibition as therapy in acute myeloid leukemia: a record of trials and tribulations. oncologist. (2011) 16:1162–74. doi: 10.1634/theoncologist.2011-0084, PMID: 21765192 PMC3228152

[65] Andre M Caobi A Miles JS Vashist A Ruiz MA Raymond AD. Diagnostic potential of exosomal extracellular vesicles in oncology. BMC cancer. (2024) 24:322. doi: 10.1186/s12885-024-11819-4, PMID: 38454346 PMC10921614

[66] Spitzberg JD Ferguson S Yang KS Peterson HM Carlson JC Weissleder R. Multiplexed analysis of EV reveals specific biomarker composition with diagnostic impact. Nat Commun. (2023) 14:1239. doi: 10.1038/s41467-023-36932-z, PMID: 36870999 PMC9985597

[67] Matthiesen R Gameiro P Henriques A Bodo C Moraes MCS Costa-Silva B. Extracellular vesicles in diffuse large b cell lymphoma: Characterization and diagnostic potential. Int J Mol Sci. (2022) 23:13327. doi: 10.3390/ijms232113327, PMID: 36362114 PMC9654702

[68] Nakamura S Yokoyama K Shimizu E Yusa N Kondoh K Ogawa M. Prognostic impact of circulating tumor DNA status post–allogeneic hematopoietic stem cell transplantation in AML and MDS. Blood J Am Soc Hematology. (2019) 133:2682–95. doi: 10.1182/blood-2018-10-880690, PMID: 30936070

[69] Xue Y Xia X Liu X Zheng Y Gu H Wang X. Applications of circulating tumor DNA in myelodysplastic syndromes and acute myeloid leukemia: promises and challenges. Front Bioscience-Landmark. (2024) 29:86. doi: 10.31083/j.fbl2902086, PMID: 38420833

[70] Boyiadzis M Whiteside TL. Plasma-derived exosomes in acute myeloid leukemia for detection of minimal residual disease: are we ready? Expert Rev Mol diagnostics. (2016) 16:623–9. doi: 10.1080/14737159.2016.1174578, PMID: 27043038 PMC5400097

[71] Döhner H Wei AH Appelbaum FR Craddock C DiNardo CD Dombret H. Diagnosis and management of AML in adults: 2022 recommendations from an international expert panel on behalf of the ELN. Blood J Am Soc Hematology. (2022) 140:1345–77. doi: 10.1182/blood.2022016867, PMID: 35797463

[72] Chen Y-F Luh F Ho Y-S Yen Y. Exosomes: A review of biologic function, diagnostic and targeted therapy applications, and clinical trials. J Biomed science. (2024) 31:67. doi: 10.1186/s12929-024-01055-0, PMID: 38992695 PMC11238361

[73] Lyu C Sun H Sun Z Liu Y Wang Q. Roles of exosomes in immunotherapy for solid cancers. Cell Death Disease. (2024) 15:106. doi: 10.1038/s41419-024-06494-z, PMID: 38302430 PMC10834551

[74] Alexiuk M Elgubtan H Tangri N. Clinical decision support tools in the electronic medical record. Kidney Int Rep. (2024) 9:29–38. doi: 10.1016/j.ekir.2023.10.019, PMID: 38312784 PMC10831391

[75] Tutrone R Donovan MJ Torkler P Tadigotla V McLain T Noerholm M. Clinical utility of the exosome based ExoDx Prostate (IntelliScore) EPI test in men presenting for initial Biopsy with a PSA 2–10 ng/mL. Prostate Cancer Prostatic Diseases. (2020) 23:607–14. doi: 10.1038/s41391-020-0237-z, PMID: 32382078 PMC7655505

[76] Mizenko RR Feaver M Bozkurt BT Lowe N Nguyen B Huang KW. A critical systematic review of extracellular vesicle clinical trials. J Extracellular Vesicles. (2024) 13:e12510. doi: 10.1002/jev2.12510, PMID: 39330928 PMC11428870

[77] Rezaie J Feghhi M Etemadi T. A review on exosomes application in clinical trials: perspective, questions, and challenges. Cell Communication Signaling. (2022) 20:145. doi: 10.1186/s12964-022-00959-4, PMID: 36123730 PMC9483361

[78] Li B Kugeratski FG Kalluri R. A novel machine learning algorithm selects proteome signature to specifically identify cancer exosomes. Elife. (2024) 12:RP90390. doi: 10.7554/eLife.90390, PMID: 38529947 PMC10965221

[79] Shin H Choi BH Shim O Kim J Park Y Cho SK. Single test-based diagnosis of multiple cancer types using Exosome-SERS-AI for early stage cancers. Nat Commun. (2023) 14:1644. doi: 10.1038/s41467-023-37403-1, PMID: 36964142 PMC10039041

[80] Chen J Zhao Z Zhu H Li X. Advances in electrochemical biosensors for the detection of tumor-derived exosomes. Front Chem. (2025) 13:1556595. doi: 10.3389/fchem.2025.1556595, PMID: 40207179 PMC11978826

[81] Lee MY Kim T-K Walters K-A Wang K. A biological function based biomarker panel optimization process. Sci Rep. (2019) 9:7365. doi: 10.1038/s41598-019-43779-2, PMID: 31089177 PMC6517383

[82] Ghosh S Rajendran RL Mahajan AA Chowdhury A Bera A Guha S. Harnessing exosomes as cancer biomarkers in clinical oncology. Cancer Cell Int. (2024) 24:278. doi: 10.1186/s12935-024-03464-5, PMID: 39113040 PMC11308730

[83] Adam G Rampášek L Safikhani Z Smirnov P Haibe-Kains B Goldenberg A. Machine learning approaches to drug response prediction: challenges and recent progress. NPJ Precis Oncol. (2020) 4:19. doi: 10.1038/s41698-020-0122-1, PMID: 32566759 PMC7296033

[84] Huang C Clayton EA Matyunina LV McDonald LD Benigno BB Vannberg F. Machine learning predicts individual cancer patient responses to therapeutic drugs with high accuracy. Sci Rep. (2018) 8:16444. doi: 10.1038/s41598-018-34753-5, PMID: 30401894 PMC6219522

[85] Qureshi R Basit SA Shamsi JA Fan X Nawaz M Yan H. Machine learning based personalized drug response prediction for lung cancer patients. Sci Rep. (2022) 12:18935. doi: 10.1038/s41598-022-23649-0, PMID: 36344580 PMC9640729

[86] Mukerjee N Bhattacharya A Maitra S Kaur M Ganesan S Mishra S. Exosome isolation and characterization for advanced diagnostic and therapeutic applications. Materials Today Bio. (2025) 31:101613. doi: 10.1016/j.mtbio.2025.101613, PMID: 40161926 PMC11950786

[87] Azenkot T Rivera DR Stewart MD Patel SP. Artificial intelligence and machine learning innovations to improve design and representativeness in oncology clinical trials. Am Soc Clin Oncol Educ Book. (2025) 45:e473590. doi: 10.1200/EDBK-25-473590, PMID: 40403202

[88] Dodig S Čepelak I Dodig M. Are we ready to integrate advanced artificial intelligence models in clinical laboratory? Biochemia Med. (2025) 35:1227091. doi: 10.11613/BM.2025.010501, PMID: 39703759 PMC11654238

[89] U.S. Food and Drug Administration (FDA). FDA proposes framework to advance credibility of AI models used for drug and biological product submissions. (2025) 2025. https://www.fda.gov/news-events/press-announcements/fda-proposes-framework-advance-credibility-ai-models-used-drug-and-biological-product-submission.

[90] St John Lynch N Loughran R McHugh M McCaffrey F. Artificial intelligence-enabled medical device standards: A multidisciplinary literature review. In: European Conference on Software Process Improvement. Cham: Springer (2024). p. 112–30.

[91] Warraich HJ Tazbaz T Califf RM. FDA perspective on the regulation of artificial intelligence in health care and biomedicine. Jama. (2025) 333:241–7. doi: 10.1001/jama.2024.21451, PMID: 39405330

[92] Yu D Li Y Wang M Gu J Xu W Cai H. Exosomes as a new frontier of cancer liquid biopsy. Mol cancer. (2022) 21:56. doi: 10.1186/s12943-022-01509-9, PMID: 35180868 PMC8855550

[93] Lyu N Hassanzadeh-Barforoushi A Rey Gomez LM Zhang W Wang Y. SERS biosensors for liquid biopsy towards cancer diagnosis by detection of various circulating biomarkers: current progress and perspectives. Nano convergence. (2024) 11:22. doi: 10.1186/s40580-024-00428-3, PMID: 38811455 PMC11136937

[94] Lai JJ Chau ZL Chen SY Hill JJ Korpany KV Liang NW. Exosome processing and characterization approaches for research and technology development. Advanced Science. (2022) 9:2103222. doi: 10.1002/advs.202103222, PMID: 35332686 PMC9130923

[95] BioSciences C. A Study of exoASO-STAT6 (CDK-004) in Patients with Advanced Hepatocellular Carcinoma (HCC) and Patients with Liver Metastases from EIther Primary Gastric Cancer or Colorectal Cancer (CRC). Cambridge, MA, USA: Codiak BioSciences (2022).

[96] Zhou B Sun X Dong B Yu S Cheng L Hu S. Antibacterial PDT nanoplatform capable of releasing therapeutic gas for synergistic and enhanced treatment against deep infections. Theranostics. (2022) 12:2580. doi: 10.7150/thno.70277, PMID: 35401821 PMC8965476

[97] Schwarz G Ren X Xie W Guo H Jiang Y Zhang J. Engineered exosomes: A promising drug delivery platform with therapeutic potential. Front Mol Biosciences. (2025) 12:1583992. doi: 10.3389/fmolb.2025.1583992, PMID: 40417062 PMC12098103

[98] Sun W Luo J-D Jiang H Duan DD. Tumor exosomes: a double-edged sword in cancer therapy. Acta Pharmacologica Sinica. (2018) 39:534–41. doi: 10.1038/aps.2018.17, PMID: 29542685 PMC5888693

[99] Galli M Magen H Einsele H Chatterjee M Grasso M Specchia G. Roneparstat (SST0001), an innovative heparanase (HPSE) inhibitor for multiple myeloma (MM) therapy: first in man study. Washington, DC: American Society of Hematology (2015).

[100] Gustave Roussy CC, Grand Paris. Phase II trial of a vaccination with tumor antigen-loaded dendritic cell-derived exosomes on patients with unresectable non-small cell lung cancer responding to induction chemotherapy. Clinicaltrials.gov (2018). https://clinicaltrials.gov/study/NCT01159288 (Accessed on 3 November 2024).

[101] Yao Y Wang C Wei W Shen C Deng X Chen L. Dendritic cells pulsed with leukemia cell-derived exosomes more efficiently induce antileukemic immunities. PloS One. (2014) 9:e91463. doi: 10.1371/journal.pone.0091463, PMID: 24622345 PMC3951359

[102] Hochhaus A Baccarani M Silver RT Schiffer C Apperley JF Cervantes F. European LeukemiaNet 2020 recommendations for treating chronic myeloid leukemia. Leukemia. (2020) 34:966–84. doi: 10.1038/s41375-020-0776-2, PMID: 32127639 PMC7214240

[103] Food U, Administration D. Public safety notification on exosome products. Food and Drug Administration (2019). https://www.fda.gov/vaccines-blood-biologics/safety-availability-biologics/public-safety-notification-exosome-products (accessed on June 1, 2025).

[104] Verma N Arora S. Navigating the global regulatory landscape for exosome-based therapeutics: challenges, strategies, and future directions. Pharmaceutics. (2025) 17:990. doi: 10.3390/pharmaceutics17080990, PMID: 40871013 PMC12389065

[105] Wong AK Mooghali M Ramachandran R Ross JS Wallach JD. Use of expedited regulatory programs and clinical development times for FDA-approved novel therapeutics. JAMA Network Open. (2023) 6:e2331753–e2331753. doi: 10.1001/jamanetworkopen.2023.31753, PMID: 37651145 PMC10472182

[106] Ronco V Dilecce M Lanati E Canonico PL Jommi C. Price and reimbursement of advanced therapeutic medicinal products in Europe: are assessment and appraisal diverging from expert recommendations? J Pharm Policy Pract. (2021) 14:30. doi: 10.1186/s40545-021-00311-0, PMID: 33741076 PMC7980570

[107] Yang D Zhang W Zhang H Zhang F Chen L Ma L. Progress, opportunity, and perspective on exosome isolation-efforts for efficient exosome-based theranostics. Theranostics. (2020) 10:3684. doi: 10.7150/thno.41580, PMID: 32206116 PMC7069071

[108] Upadhya D Shetty AK. MISEV2023 provides an updated and key reference for researchers studying the basic biology and applications of extracellular vesicles. Stem Cells Trans Med. (2024) 13:848–50. doi: 10.1093/stcltm/szae052, PMID: 39028333 PMC11386207

[109] Qiu L Liu X Zhu L Luo L Sun N Pei R. Current advances in technologies for single extracellular vesicle analysis and its clinical applications in cancer diagnosis. Biosensors. (2023) 13:129. doi: 10.3390/bios13010129, PMID: 36671964 PMC9856491

[110] Omrani M Beyrampour-Basmenj H Jahanban-Esfahlan R Talebi M Raeisi M Serej ZA. Global trend in exosome isolation and application: an update concept in management of diseases. Mol Cell Biochem. (2024) 479:679–91. doi: 10.1007/s11010-023-04756-6, PMID: 37166542 PMC10173230

[111] Shaman JA. The future of pharmacogenomics: integrating epigenetics, nutrigenomics, and beyond. J Personalized Med. (2024) 14:1121. doi: 10.3390/jpm14121121, PMID: 39728034 PMC11677977

[112] Aries A Drénou B Lahlil R. Liquid biopsy and epigenetic signatures in AML, ALL, and CNS tumors: diagnostic and monitoring perspectives. Int J Mol Sci. (2025) 26:7547. doi: 10.3390/ijms26157547, PMID: 40806675 PMC12346961

[113] Li W Zhou XJ. Methylation extends the reach of liquid biopsy in cancer detection. Nat Rev Clin Oncol. (2020) 17:655–6. doi: 10.1038/s41571-020-0420-0, PMID: 32732909 PMC8284425

[114] Ma L Guo H Zhao Y Liu Z Wang C Bu J. Liquid biopsy in cancer: current status, challenges and future prospects. Signal Transduction Targeted Ther. (2024) 9:336. doi: 10.1038/s41392-024-02021-w, PMID: 39617822 PMC11609310

[115] Nikanjam M Kato S Kurzrock R. Liquid biopsy: current technology and clinical applications. J Hematol Oncol. (2022) 15:131. doi: 10.1186/s13045-022-01351-y, PMID: 36096847 PMC9465933

[116] Zhao Y O’Keefe CM Hu J Allan CM Cui W Lei H. Multiplex digital profiling of DNA methylation heterogeneity for sensitive and cost-effective cancer detection in low-volume liquid biopsies. Sci Adv. (2024) 10:eadp1704. doi: 10.1126/sciadv.adp1704, PMID: 39576863 PMC11584010

[117] Pandey S Yadav P. Liquid biopsy in cancer management: Integrating diagnostics and clinical applications. Pract Lab Med. (2025) 43:e00446. doi: 10.1016/j.plabm.2024.e00446, PMID: 39839814 PMC11743551

[118] Kustanovich A Schwartz R Peretz T Grinshpun A. Life and death of circulating cell-free DNA. Cancer Biol Ther. (2019) 20:1057–67. doi: 10.1080/15384047.2019.1598759, PMID: 30990132 PMC6606043

